# Characteristics and management of systemic sclerosis-related osteomyelitis: a retrospective cohort study

**DOI:** 10.1007/s00296-025-05815-5

**Published:** 2025-03-12

**Authors:** Toshiki Miwa, Koh Okamoto, Hayakazu Sumida, Satoshi Toyama, Shinichi Sato, Takeya Tsutsumi

**Affiliations:** 1 Department of Infectious Diseases, The University of Tokyo Hospital, Tokyo, Japan; 2 Department of Infectious Diseases, Graduate School of Medical and Dental Sciences, Institute of Science Tokyo, 1-5-45 Yushima, Bunkyo-ku, Tokyo, 113-8519 Japan; 3 Department of Dermatology, Graduate School of Medicine, The University of Tokyo, 7-3-1 Hongo, Bunkyo-ku, Tokyo, 113-8655 Japan; 4 Scleroderma Center, The University of Tokyo Hospital, Tokyo, Japan; 5 SLE Center, The University of Tokyo Hospital, Tokyo, Japan

**Keywords:** Systemic sclerosis, Osteomyelitis

## Abstract

Digital ulcers in patients with systemic sclerosis (SSc) can be complicated by SSc-related osteomyelitis (SRO). The microbiological data and optimal management of SRO remain unclear. This single-center retrospective study involved patients with SSc aged 18 or older from April 2005 to March 2022. Diagnosis of SRO was based on clinical presentation and MRI findings. The accuracy of the superficial swab culture results was estimated using the bone culture as a reference. Temporal changes in local signs for up to a year were collected, and their association with (1) duration of antimicrobial therapy (> 6 weeks) or (2) surgical interventions was assessed using univariable analyses. Among the 2,126 patients, 46 (2.2%) were diagnosed with SRO. In seven patients whose swab and bone cultures were both available, two (28.6%) had swab cultures identifying all the organisms detected in bone cultures. Resolution of local inflammatory signs consistently preceded wound closure. Three months after therapy initiation, prolonged antimicrobial therapy was not significantly associated with the resolution of local inflammatory signs (16/19 [84.2%] vs. 12/14 [85.7%]; *P* = 1.00), and surgical intervention was not significantly associated with wound dehiscence (6/9 [66.7%] vs. 20/24 [83.3%]; *P* = 0.36). Superficial swab cultures may not reliably reflect the true causative organism of SRO. Prolonging antimicrobial therapy beyond six weeks may be of little benefit for patients with SRO when local inflammatory signs improve. Surgical intervention may be a safe and effective option for selected patients with SRO.

**Supplementary Information:**

The online version contains supplementary material available at 10.1007/s00296-025-05815-5.

## Introduction

Systemic sclerosis (SSc) is an autoimmune disease characterized by vascular, inflammatory, and fibrotic dysfunction that affects multiple organ systems [1]. Digital ulcers are common clinical manifestations of SSc, and their prevalence among patients with SSc is 15–20% [2, 3]. The pathogenesis of SSc-related digital ulcers is multifactorial and includes vascular compromise, contracture of fingers, and calcinosis [4]. Digital ulcers are a hallmark of SSc; they are refractory to local therapy, resulting in chronic pain and functional impairment, and negatively affect the quality of life of patients [4]. Osteomyelitis is a serious complication of digital ulcers [2, 5, 6].

SSc-related osteomyelitis (SRO) shares partial characteristics with diabetic foot osteomyelitis (DFO), which also complicates ulcers in the extremities and is often treated with prolonged antimicrobial therapy with or without surgical interventions [2, 7]. However, there are some unresolved issues regarding SRO management. First, and most importantly, similar to DFO [8], treatment success in guiding antimicrobial therapy is ill-defined. These infections are locally formed, and local signs, such as preexisting ulcers, may persist regardless of the resolution of inflammation [8]. This is in contrast to vertebral osteomyelitis, in which the resolution of both systemic and local inflammatory signs is used to define a cure [9]. Second, compared to DFO [10], microbiological data on deep tissue specimens, which have important implications in choosing antimicrobials, are scarce for SRO. Deep-tissue sampling is rarely performed in SRO [7, 11]. Although bone culture and biopsy are the gold standards for diagnosing osteomyelitis, clinical and imaging findings are often used as substitutes [11, 12]. Third, unlike in DFO, aggressive surgical interventions such as amputation are often averted in SRO because of vasculopathy, and its role remains uncertain [4]. To address these gaps, we aimed to explore the definition of treatment success and optimal management of patients with SRO at a reference center for SSc.

The objectives of our study were to (1) describe the changes in clinical symptoms, signs, and laboratory data before and after treatment to explore the criteria for treatment success in patients with SRO; (2) evaluate the concordance between superficial and deep tissue cultures; and (3) investigate the role of prolonged antimicrobial and surgical therapy.

## Methods

### Study design, setting, and participants

This single-center retrospective study was conducted at The University of Tokyo Hospital (UTH) in Japan. UTH has a Scleroderma Center comprising a multidisciplinary team that provides comprehensive care to patients with SSc [13]. We included all individuals with positive SSc-related autoantibody test results who were 18 years or older during the study period from April 2005 to March 2022 at UTH. SSc-related autoantibodies include anti-centromere, anti-topoisomerase I (Scl-70), anti-RNA polymerase III, and anti-U1-ribonucleoprotein (RNP) antibodies [1] and have been consistently measured in patients at our referral center with clinical suspicion of SSc.

In this study, SRO was diagnosed as per the following criteria: (1) the patient fulfilled the 2013 American College of Rheumatology (ACR) /European League Against Rheumatism (EULAR) classification criteria of SSc [14]; (2) the patient had clinical presentations suspicious for osteomyelitis of the extremities at the same time or after receiving the diagnosis of SSc; (3) the patient underwent magnetic resonance imaging (MRI) of the extremities; and (4) MRI findings were consistent with osteomyelitis. Using the ACR/EULAR SSc criteria in 2013 [14], we excluded patients with anti-U1-RNP antibodies who had mixed connective tissue disease in the absence of SSc. At UTH, treating physicians consistently ordered MRI for patients suspected of having osteomyelitis unless they had a contraindication for MRI. Patients with osteomyelitis at other sites such as vertebral osteomyelitis were excluded. If the patients experienced SRO more than once during the study period, only the first episode was included in the analyses. After a diagnosis of osteomyelitis was made based on clinical and MRI findings, the patients received therapy in either an inpatient or outpatient setting. Infectious disease (ID) consultations were available at the request of the treating physician.

### Variables, data sources, and measurement

We collected the baseline characteristics of the study participants, including demographics, comorbidities, medications, clinical manifestations, autoantibodies, laboratory, microbiological, and pathologic data, and imaging findings through electronic health records. The timing of SSc diagnosis was ascertained according to documentation in the medical records, and the time of osteomyelitis diagnosis was determined as the day of the index MRI. We also collected data on ID consultations, duration of antimicrobial therapy, and surgical interventions. Surgical interventions included (1) amputation and (2) non-amputation-surgical interventions, including aspiration and drainage of deep tissue. Given the poor definition of treatment success for SRO, treatment outcomes in our study included mortality, white blood cell counts, C-reactive protein levels, and local signs (i.e., gangrene, redness, swelling, warmth, pus drainage, and wound dehiscence) at 3, 6, and 12 months after the initiation of treatment and these details were retrieved from the medical records.

### Statistical analyses

First, to assess the accuracy of the microbiological diagnosis, we calculated the sensitivity and specificity of superficial swab cultures using bone culture as a standard test. Second, to explore the useful markers for treatment success, we evaluated the agreement of post-treatment improvement between local signs and symptoms and C-reactive protein levels using kappa statistics. Third, univariable analyses were performed to assess the association between treatment outcomes and (1) prolonged antimicrobial therapy (i.e., duration > 42 days) or (2) surgical interventions. In this analysis, we did not conduct a multivariable analysis due to the small sample size. Instead, we incorporated comparisons of confounders, such as medications for digital ulcers and systemic immunosuppressants between the groups, to illustrate the patient background. The Chi-squared test, Fisher’s exact test, or Mann-Whitney U test were performed as appropriate, with statistical significance defined as a two-tailed *P* < 0.05. For the analysis of SRO treatment, we excluded patients who were followed up without systemic antimicrobial therapy. We performed either the Fisher’s exact test or the chi-squared test for categorical variables, as appropriate, using Stata version 16 software (StataCorp, College Station, TX, USA).

### Patient consent statement

This study was approved by the Institutional Review Board of The University of Tokyo Hospital (approval number 2023107NI, approval day September 11, 2023). Given the retrospective nature of the study, the requirement for patient consent was waived in accordance with the Declaration of Helsinki. This study was performed in accordance with Strengthening the Reporting of Observational Studies in Epidemiology (STROBE) guidelines.

## Results

### Baseline characteristics of the study population

**Fig. 1:**
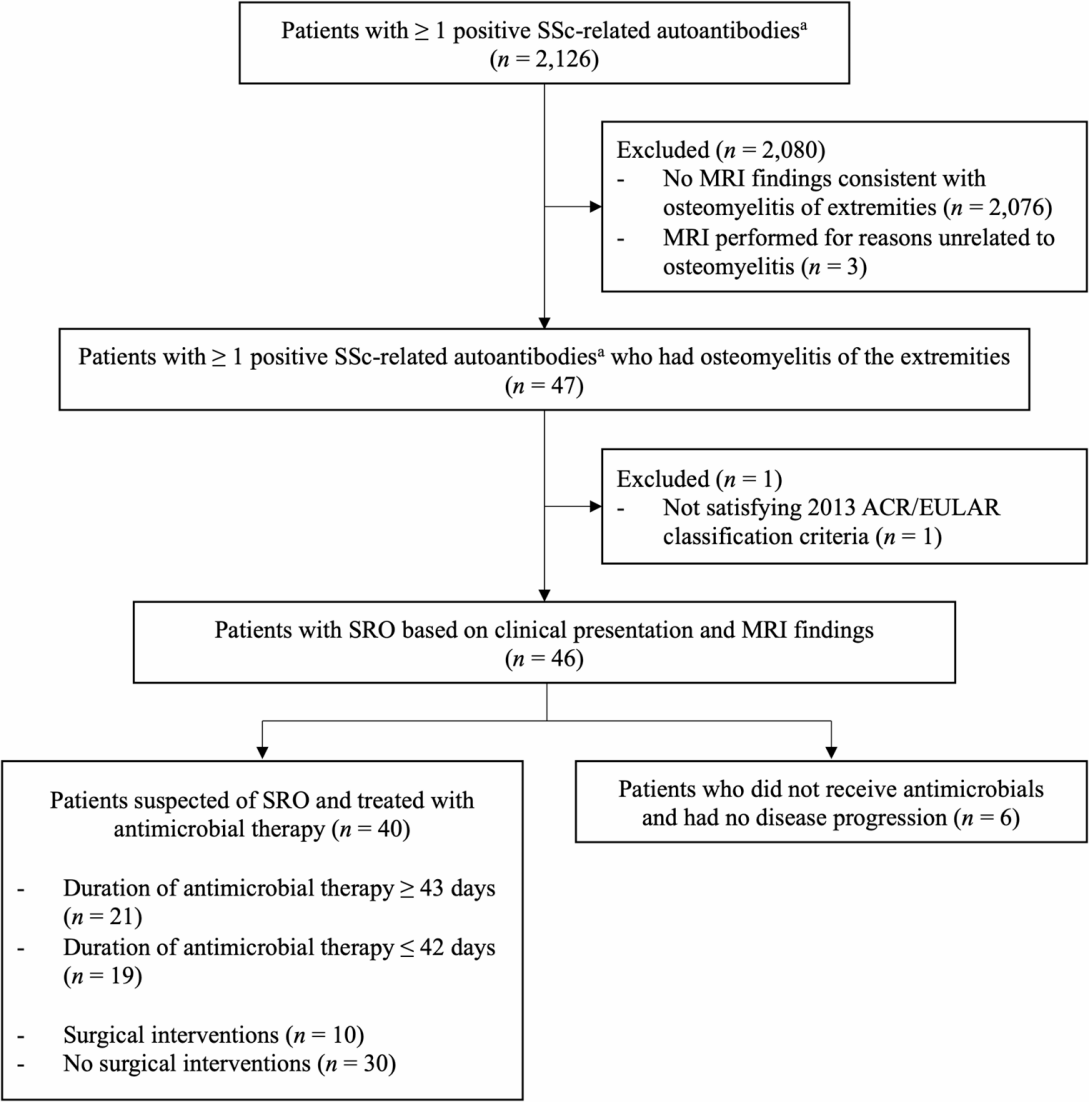
Flow diagram of study participants. Note: Abbreviations: SSc, systemic sclerosis; SRO, systemic sclerosis-related osteomyelitis; ACR, American College of Rheumatology; EULAR, European League Against Rheumatism. ^a^SSc-related autoantibodies included anti-centromere antibodies, anti-topoisomerase I (Scl-70) antibodies, anti-RNA polymerase III antibodies, and anti-U1-ribonucleoprotein (RNP) antibodies

During the study period, 2,126 patients tested positive for at least one SSc-related autoantibody, 50 (2.4%) of whom had features of osteomyelitis in the MRI of the extremities. The chart review confirmed that three patients (3/50,6.0%) underwent MRI for arthritis, digital ischemia, and dactylitis and did not have clinical presentations suspicious for osteomyelitis of the extremities. Thus, 47 patients (47/2,126:2.2%) with positive SSc-related autoantibodies developed osteomyelitis of the extremities. After excluding one patient who did not satisfy the 2013 ACR/EULAR classification criteria, we identified 46 patients with SRO. In total, 49 episodes of SRO occurred, and the first episode in each patient was included in the present study (Fig. 1). The median age at the development of SRO was 66.5 years (interquartile range: 54.5–77.3 years), and 44 participants (95.7%) were female. Eleven (23.9%) participants were past or current smokers. Regarding the type of SSc, 18 (39.1%) had diffuse cutaneous SSc, and 18 had limited cutaneous SSc. The median modified Rodnan total skin thickness score was 5.5 (interquartile score: 2.0-14.3) [15], and 28 patients (60.9%) were on immunosuppressive therapy (Table 1). Among the overall cohort of 2,126 patients with SSc-related autoantibodies, those who developed osteomyelitis of the extremities were more likely to have anti-topoisomerase I antibodies than those who did not (36.2% vs. 19.8%, respectively; *P* < 0.01) (Online Resource 1).

**Table 1:** Baseline characteristics of patients with systemic sclerosis-related osteomyelitis at enrollment

Variables	Total (*n* = 46)
**Patient demographics**	
Age, median, (IQR) years	66.5 (54.5–77.3)
Female sex	44 (95.7)
History of smoking	11 (23.9)
**Characteristics of systemic sclerosis**	
Cutaneous subset	
Diffuse cutaneous systemic sclerosis	18 (39.1)
Limited cutaneous systemic sclerosis	18 (39.1)
Unidentified	10 (21.7)
Autoantibodies	
Anti-centromere antibody	25 (54.3)
Anti-topoisomerase I antibody	17 (37.0)
Anti-U1RNP antibody	13 (28.3)
Anti-RNA polymerase III antibody	1 (2.2)
Modified Rodnan total skin thickness score, median, (IQR) (*n* = 30)^b^	5.5 (2-14.3)
Digital ulcers (*n* = 45)^b^	43 (95.6)
Raynaud’s phenomenon (*n* = 40)^b^	38 (95.0)
Pitting scars (*n* = 24)^b^	19 (79.2)
Telangiectasia (*n* = 29)^b^	20 (69.0)
Nail fold bleeding (*n* = 34)^b^	20 (58.8)
**Comorbidities**	
Gastroesophageal reflux disease	27 (58.7)
Interstitial lung disease	20 (43.5)
Ischemic heart disease	12 (26.1)
Pulmonary hypertension^a^	8 (17.4)
Scleroderma renal crisis	2 (4.3)
Hypertension	14 (30.4)
Peripheral artery disease	12 (26.1)
Chronic kidney disease	14 (30.4)
History of malignancy	6 (13.0)
Cerebrovascular disease	5 (10.9)
Diabetes	4 (8.7)
Stasis dermatitis	1 (2.2)
Other collagen vascular diseases	
Sjögren syndrome	8 (17.4)
Systemic lupus erythematosus	6 (13.0)
Rheumatoid arthritis	3 (6.5)
Antiphospholipid syndrome	3 (6.5)
Vasculitis	2 (4.3)
Polymyositis/dermatomyositis	2 (4.3)
**Medications**	
Non-immunosuppressants	
Proton pump inhibitors	46 (100.0)
Prostacyclin analogs	30 (65.2)
Endothelin receptor antagonists	24 (52.2)
Serotonin receptor antagonists	19 (41.3)
Phosphodiesterase inhibitors	18 (39.1)
Calcium channel blockers	15 (32.6)
Warfarin	7 (15.2)
Direct oral anticoagulants	3 (6.5)
Nitrates	2 (4.3)
Soluble guanylate cyclase agonists	2 (4.3)
Any immunosuppressants	28 (60.9)
Glucocorticoids	27 (58.7)
Calcineurin inhibitors	3 (6.5)
Mizoribine	3 (6.5)
Mycophenolate mofetil	2 (4.3)
Intravenous immunoglobulin	2 (4.3)
Azathioprine	1 (2.2)
Methotrexate	1 (2.2)
Intravenous cyclophosphamide	1 (2.2)
Rituximab	1 (2.2)
Tocilizumab	1 (2.2)

Note: Data are presented as a number (%) unless otherwise specified

Abbreviations: IQR, interquartile range

^a^Pulmonary hypertension was defined as either mean pulmonary artery pressure ≥ 20mmHg measured by right heart catheterization or right ventricular systolic pressure ≥ 50mmHg measured by cardiac echography

^b^Some data were missing in the health record

### Clinical manifestations and laboratory and microbiological characteristics of SRO

The time between the diagnosis of SSc and SRO varied considerably, ranging from 251 days to 35 years; 35 (76.1%) of the 46 study participants were diagnosed with SRO five years after the diagnosis of SSc. Twenty-two patients (47.8%) had toe osteomyelitis and 21 (45.7%) had finger osteomyelitis. The most common manifestation was local gangrene (*n* = 33, 71.7%), followed by local redness (*n* = 22, 47.8%), and local swelling (*n* = 19, 41.3%) (Online Resource 2). Only 11 patients (23.9%) had fever. The median white blood cell counts and C-reactive protein (CRP) level at the diagnosis of osteomyelitis were 5.9$\:\:\times\:$10^3^/µL (normal range: 3.3–8.6$\:\:\times\:$10^3^/ µL) and 0.40 mg/dL (normal range: 0.0–0.3 mg/dL), respectively. Bone biopsy was performed in eight patients (17.4%), of whom seven (87.5%) had a pathological diagnosis of osteomyelitis and one (12.5%) had an indeterminate result (Table 2).

**Table 2:** Clinical, laboratory, and imaging characteristics of systemic sclerosis-related osteomyelitis

Variables	Total (*n* = 46)
**Clinical manifestations**	
Time from the diagnosis of systemic sclerosis to osteomyelitis	
Within 1 year	4 (8.7)
1–5 year	7 (15.2)
More than 5 years	35 (76.1)
Infection site	
Toe	22 (47.8)
Finger	21 (45.7)
Foot	3 (6.5)
Fever	11 (23.9)
Local gangrene	33 (71.7)
Local redness	22 (47.8)
Local swelling	19 (41.3)
Local warmth	14 (30.4)
Pus drainage	13 (28.3)
Bone exposure	9 (19.6)
**Laboratory data at enrollment**	
White blood cells level, median, (IQR) $\:\times\:$10^3^/µL	5.9 (4.7–8.1)
CRP level, median, (IQR) mg/dL	0.40 (0.11–1.43)
**Imaging studies**	
CT findings consistent with osteomyelitis (*n* = 4)	1 (25.0)
**Cultures**	
Positive blood cultures (*n* = 11)	3 (27.3)
Positive superficial swab cultures (*n* = 36)	31 (86.1)
Positive cultures of deep tissue other than bone (*n* = 12)	10 (83.3)
Positive bone culture (*n* = 8)	8 (100.0)
**Pathology**	
Bone histopathology consistent with osteomyelitis (*n* = 8)	7 (87.5)

Note: Abbreviations: IQR, interquartile range; CRP, C-reactive protein; CT, computerized tomography

Regarding microbiological testing, blood cultures were drawn from 11 patients (23.9%), and three (6.5%) had bacteremia. Superficial swabs and bone cultures were submitted in 36 (78.2%) and eight (17.4%) patients, respectively, and the proportions of positive results were 86.1% (*n* = 31) and 100% (*n* = 8), respectively (Table 3). Bone cultures from eight patients showed that the most common organism was *Staphylococcus aureus* (*n* = 5, 62.5%). *Pseudomonas aeruginosa* was the second most commonly detected organism in superficial swab cultures (*n* = 4, 11.4%), followed by *Enterococcus faecalis* and *Escherichia coli* but was not detected in bone cultures. In seven patients who underwent both swab and bone cultures, the overall sensitivity of swab cultures was 71.4% for detecting at least one species and 28.6% for detecting all species from bone cultures.

**Table 3:** The sensitivity and specificity of superficial swab culture for bone culture on microbiological results

Organisms	Bone culture (*n* = 8)^a^	Swab culture (*n* = 36)	Validity of swab culture^b^	
			sensitivity	specificity
**Gram-positive organisms**				
*Staphylococcus aureus*	5 (62.5)	19 (52.8)	75.0%	66.7%
*Streptococcus* spp.	1 (12.5)	3 (8.3)	0%	0%
*Enterococcus faecalis*	1 (12.5)	4 (11.4)	100%	100%
**Gram-negative organisms**				
* Escherichia coli*	1 (12.5)	4 (11.4)	100%	100%
*Pseudomonas aeruginosa*	0	5 (13.9)	0%	0%
**Obligate anaerobes**				
*Bacteroides* spp.	1 (12.5)	2 (5.6)	100%	100%
**Total**	8 (100)	31 (86.1)		
Detection of ≥ 1 species from bone culture			71.4%	N/A^c^
Detection of all species from bone culture			28.6%	N/A^c^

Note: Data are presented as a number (%) unless otherwise specified

Abbreviations: N/A, not applicable

^a^Organisms recoverd from bone culture included *Moraxella species* (*n* = 1), *Peptoniphilus harei* (*n* = 1), *Proteus hauseri* (*n* = 1), and *Candida glabrata* (*n* = 1)

^b^Validity was calculated using data in seven participants who examined both bone and swab culture

^c^Specificity was unavailable because all bone cultures were positive

### Subsequent clinical course of patients not receiving antimicrobial therapy

Of the 46 study participants who were initially suspected of having osteomyelitis clinically, six (13.0%) were followed up without antimicrobial therapy. One patient was in the end-of-life stage. Another patient remained stable without antimicrobial therapy after confirming the negativity of bone culture. The other four patients did not receive antimicrobial therapy because of the subsequent spontaneous improvement of local signs and symptoms. No patient developed a relapse during the follow-up period.

### Management and post-treatment clinical course of SRO

Among the study participants, 40 (87.0%) received antimicrobial therapy and were left for further analysis. The median duration of antimicrobial therapy was 47.5 days (interquartile range: 26.5–83.5 days). An ID consultation within 7 days of SRO diagnosis was performed in 28 patients (62.2%). Four patients (10.0%) underwent amputation, six (15.0%) underwent non-amputation-surgical interventions, and 30 (75.0%) received antimicrobial therapy alone.

Local inflammatory signs improved over time, with 84.8% and 100% of the patients showing no local signs or symptoms at three months and 12 months, respectively. Although the proportion of remaining open wounds also decreased gradually, 32.1% (9/28) of the patients still had open wounds after 12 months. In 23 patients with initially elevated CRP levels, the agreement between the subsequent reduction in CRP levels and the improvement of these local findings was largely poor, with the kappa statistics ranging from 0 to 0.31 (Online Resource 3). Five patients (12.5%) died within the follow-up period of one year after the diagnosis of SRO, of whom one died of progressive heart failure while receiving antimicrobial therapy for SRO. The other four died of SRO-unrelated causes, such as acute coronary syndrome. When comparing short course (≤ 42 days) vs. prolonged antimicrobial therapy (≥ 43 days), there was no statistically significant difference in the proportion of patients receiving surgical interventions or residual local manifestations (Table 4).

**Table 4:** The management and outcomes of study participants receiving antimicrobial therapy in patients with systemic sclerosis-related osteomyelitis

Variables	Total (*n* = 40)	Antimicrobial therapy		*P* value
		Duration ≤ 42 days (*n* = 19)	Duration ≥ 43 days (*n* = 21)	
**Underlying medication use**				
Prostacyclin analogs	27 (67.5)	12 (63.2)	15 (71.4)	0.58
Endothelin receptor antagonists	21 (52.5)	10 (52.6)	11 (52.4)	0.99
Phosphodiesterase inhibitors	16 (40.0)	5 (31.3)	11 (52.4)	0.09
Serotonin receptor antagonists	15 (37.5)	7 (36.8)	8 (38.1)	0.93
Calcium channel blockers	13 (32.5)	8 (42.1)	5 (23.8)	0.22
Any immunosuppressants	26 (65.0)	12 (63.2)	14 (66.7)	0.82
**Management**				0.68
Amputation in addition to systemic antimicrobial therapy	4 (10.0)	1 (5.3)	3 (14.3)	
Surgical debridement in addition to systemic antimicrobial therapy	6 (15.0)	3 (15.8)	3 (7.0)	
Systemic antimicrobial therapy alone	30 (75.0)	16 (84.2)	14 (66.7)	
**Outcomes** ^a^				
Resolution of local inflammation^b^				
3 months after the initiation of treatment	28/33 (84.8)	12/14 (85.7)	16/19 (84.2)	1.00
6 months after the initiation of treatment	31/32 (96.9)	12/13 (92.3)	19/19 (100.0)	
12 months after the initiation of treatment	28/28 (100.0)	12/12 (100.0)	16/16 (100.0)	
Wound closure				
3 months after the initiation of treatment	7/33 (21.2)	4/14 (28.6)	3/19 (15.8)	0.42
6 months after the initiation of treatment	13/32 (40.6)	5/13 (38.5)	8/19 (42.1)	1.00
12 months after the initiation of treatment	19/28 (67.9)	9/12 (75.0)	10/16 (62.5)	0.69

Note: Data are presented as a number (%) unless otherwise specified

Abbreviations: IQR, interquartile range

^a^Outcome data of patients who died (*n* = 5) or were transferred to other hospitals during the follow-up period (*n* = 7) were missing

^b^Signs of local inflammation include gangrene, redness, swelling, warmth, and pus drainage

The reasons for undergoing surgical intervention in 10 patients (10/40, 25.0%) included refractoriness to antimicrobial therapy (*n* = 7), abscess formation (*n* = 2), and a lower likelihood of subsequent re-epithelization (*n* = 1). Although not statistically significant, those who underwent surgical intervention tended to have wound closure earlier than others (Table 5).

**Table 5:** The association between surgical therapy and the duration of antimicrobial therapy and clinical outcomes in patients with systemic sclerosis-related osteomyelitis

Variables	Surgical intervention (*n* = 10)	No surgical intervention (*n* = 30)	*P* value
**Underlying medication use**			
Prostacyclin analogs	7 (70.0)	20 (66.7)	0.85
Endothelin receptor antagonists	6 (60.0)	15 (50.0)	0.58
Phosphodiesterase inhibitors	6 (60.0)	10 (33.3)	0.14
Serotonin receptor antagonists	2 (20.0)	13 (43.3)	0.19
Calcium channel blockers	1 (10.0)	12 (40.0)	0.08
Any immunosuppressants	4 (40.0)	22 (73.3)	0.06
**Management**			
Duration of antimicrobial therapy within a year of the diagnosis, (IQR) days^a^	42 (35–76)	47.5 (14-93.8)	0.45
**Outcomes** ^**b**^			
Resolution of local inflammation^c^			
3 months after the initiation of treatment	7/9 (77.8)	21/24 (87.5)	0.60
6 months after the initiation of treatment	8/9 (88.9)	23/23 (100.0)	
12 months after the initiation of treatment	6/6 (100.0)	22/22 (100.0)	
Wound closure			
3 months after the initiation of treatment	3/9 (33.3)	4/24 (16.7)	0.36
6 months after the initiation of treatment	5/9 (55.6)	8/23 (34.8)	0.43
12 months after the initiation of treatment	5/6 (83.3)	14/22 (63.6)	0.63

Note: Data are presented as a number (%) unless otherwise specified

Abbreviations: IQR, interquartile range

^a^Longer than one year-course of antimicrobial therapy was calculated as 365 days

^b^Outcome data of patients who died (*n* = 5) or were transferred to other hospitals during the follow-up period (*n* = 7) were missing

^c^Signs of local inflammation include gangrene, redness, swelling, warmth, and pus drainage

## Discussion

In this retrospective study, we identified 47 patients with osteomyelitis among > 2,000 patients with positive SSc-related autoantibodies. Both local inflammatory signs and wound dehiscence resolved over time in many patients receiving therapy. However, wound closure followed the resolution of local signs, which suggests the improvement of local inflammation, among others, as a useful clinical criterion for treatment success. The sensitivity and specificity of superficial swab cultures for bone cultures were not consistently high. The duration of the antimicrobial therapy was highly variable. Surgical interventions were performed infrequently; however, no association between surgical intervention and delayed wound healing was demonstrated. These findings may guide the diagnostic and therapeutic approach to SRO, where relevant previous reports are scarce.

Similar to DFO [8], the absence of a definition of treatment success may be a major obstacle to exploring an appropriate approach for SRO. Potential surrogate markers include the resolution of local inflammation, wound healing, improvement of biomarkers, or a combination of these factors [8]. We collected serial data on these factors up to a year later, revealing that the signs of local inflammation and wound dehiscence improved over time and that the resolution of local inflammation preceded wound healing. In contrast, inflammatory biomarkers such as white blood cell counts and C-reactive protein levels were mostly within or near the normal range even before treatment, presumably because of the local inflammatory nature of SRO. These findings suggest that improvement in local inflammatory signs, rather than blood biomarkers, may be a good indicator of SRO treatment success.

There were some interesting findings regarding the microbiological and antimicrobial therapies of SRO in this study. First, *S. aureus* was the most common causative organism based on bone culture. This is consistent with the results of previous studies, although the microbiological data in these studies were based on superficial swab cultures [7, 16]. Second, the true involvement of *P*. *aeruginosa* in deep tissue was not common, in contrast to superficial swab culture results. Empiric antimicrobial therapy with antipseudomonal coverage can be reserved for selected patients with chronic digit osteomyelitis, depending on local epidemiology [17]. In addition, similar to DFO [10], superficial swab cultures may be of little value in estimating the true causative organisms of SRO, which supports the importance of evaluating deep tissue cultures before empirically escalating antimicrobial coverage when patients with SRO do not respond to the initial antimicrobial therapy. Third, prolonged antimicrobial therapy was not associated with improved local symptoms or wound healing. The benefit of antimicrobial therapy for longer than six weeks has not been established in DFO [18, 19] and prolonged antimicrobial therapy may not be necessary for SRO.

The need for surgical intervention for chronic osteomyelitis remains controversial. Observational studies have reported that 17–97% of patients with DFO were successfully treated with medical therapy alone, despite the presence of confounding by indication [8, 20]. Similarly, in this study on SRO, improvement of local inflammatory signs was documented in nearly 70% of the patients treated with medical therapy alone. The SRO-specific reason for avoiding surgery may be the concern that ischemic and fibrotic changes in soft tissue related to SSc hamper the natural wound-healing process [4]. However, surgery was not associated with wound dehiscence in this study. Instead, patients who underwent surgical intervention tended to have earlier wound closure than those who received medical therapy alone. Consistent with recommendations in previous narrative reviews [21, 22], surgical intervention may be an option in selected patients with SRO, especially those who have an insufficient response to initial antimicrobial therapy.

The prevalence of SRO among patients with SSc in this study was 2.2%, which is lower than that reported in previous studies (7.7–15.8%) [5–7]. It is impossible to compare these numbers directly because the definitions of SRO differ. Nevertheless, given the genetic predisposition to SSc [23], differences in ethnicity may partially explain the differences in the prevalence of SRO. Furthermore, recent progress in SSc pharmacotherapy such as the advent of endothelin receptor antagonists, may affect the development of digital ulcers [24], thereby preventing SRO. In addition, the proportion of patients with anti-topoisomerase I antibodies was higher among those with osteomyelitis than among those without osteomyelitis, in line with a previous study [7]. However, these aspects require further investigation.

The present study had certain limitations. First, the study size was relatively small, owing to the low prevalence of osteomyelitis in our cohort and the lack of statistical power to address the associations between the treatment strategy and subsequent outcomes. Likewise, bone culture tests were infrequently performed, warranting further studies to determine the precise sensitivity and specificity of superficial swab cultures. However, we identified patients with SRO among over 2,000 patients with SSc, which was larger than that reported in previous studies [5, 7]. Second, SRO was defined based on clinical presentation and MRI findings, and very few patients had pathological confirmation. However, although pathological confirmation is generally preferred [12], deep-tissue diagnoses are infrequently available in patients suspected of having SRO, as seen in this study. To reduce the chance of false positivity in the MRI findings, we ascertained that the patients had clinical presentations compatible with SRO. Third, the study findings related to clinical outcomes need to be interpreted with caution as there may be confounding factors, which are inherent to the retrospective nature of the study. Future studies with a large sample size are warranted to determine the optimal duration of antimicrobial therapy and to identify the population that would benefit the most from surgical interventions. Fourth, the microbiological epidemiology of the causative organisms and their resistance may be center-specific, although our findings are largely consistent with those of previous studies on other types of chronic osteomyelitis [10, 17].

In summary, the diagnostic and management approaches for SRO are highly heterogeneous, probably due to the lack of major guidelines. Response to antimicrobial therapy should be evaluated based on improvements in local inflammation, and prolonged therapy may not be necessary. For patients who do not respond to medical therapy, obtaining a deep tissue culture to identify the true causative organisms and surgical intervention may be safe and effective options in selected cases. While more research is necessary, these measures may be the first step toward the optimal management of SRO.

## Electronic supplementary material

Below is the link to the electronic supplementary material.

Supplementary Material 1

Supplementary Material 2

Supplementary Material 3

Supplementary Material 4

## Data Availability

Data are not publicly available due to ethical considerations but are available from the corresponding author upon reasonable request.

## References

[1] Volkmann ER, Andréasson K, Smith V (2023) Systemic sclerosis. Lancet 401(10373):304–318. 10.1016/s0140-6736(22)01692-0 36442487 10.1016/S0140-6736(22)01692-0 PMC9892343

[2] Cappelli L, Wigley FM (2015) Management of Raynaud Phenomenon and Digital Ulcers in Scleroderma. Rheum Dis Clin North Am 41(3):419–438. 10.1016/j.rdc.2015.04.005 26210127 10.1016/j.rdc.2015.04.005

[3] Muangchan C, Baron M, Pope J (2013) The 15% rule in scleroderma: the frequency of severe organ complications in systemic sclerosis. A systematic review. J Rheumatol 40(9):1545–1556. 10.3899/jrheum.121380 23858045 10.3899/jrheum.121380

[4] Steen V, Denton CP, Pope JE, Matucci-Cerinic M (2009) Digital ulcers: overt vascular disease in systemic sclerosis. Rheumatology (Oxford) 48 Suppl 3:iii19-24. 10.1093/rheumatology/kep105 10.1093/rheumatology/kep105 19487218

[5] Giuggioli D, Manfredi A, Lumetti F, Colaci M, Ferri C (2018) Scleroderma skin ulcers definition, classification and treatment strategies our experience and review of the literature. Autoimmun Rev 17(2):155–164. 10.1016/j.autrev.2017.11.020 29196241 10.1016/j.autrev.2017.11.020

[6] Yayla ME, Yurteri EU, Torgutalp M, Eroğlu D, Sezer S, Dinçer ABK, Gülöksüz EGA, Yüksel ML, Yılmaz R, Ateş A, Turgay TM, Kınıklı G (2022) Causes of severe infections in patients with systemic sclerosis and associated factors. Turk J Med Sci 52(6):1881–1888. 10.55730/1300-0144.5535 36945989 10.55730/1300-0144.5535 PMC10390190

[7] Giuggioli D, Manfredi A, Colaci M, Lumetti F, Ferri C (2013) Osteomyelitis complicating scleroderma digital ulcers. Clin Rheumatol 32(5):623–627. 10.1007/s10067-012-2161-7 23307325 10.1007/s10067-012-2161-7

[8] Truong DH, Bedimo R, Malone M, Wukich DK, Oz OK, Killeen AL, Lavery LA (2022) Meta-analysis: outcomes of Surgical and Medical Management of Diabetic Foot Osteomyelitis. Open Forum Infect Dis 9(9):ofac407. 10.1093/ofid/ofac407 36147596 10.1093/ofid/ofac407 PMC9487605

[9] Bernard L, Dinh A, Ghout I, Simo D, Zeller V, Issartel B, Le Moing V, Belmatoug N, Lesprit P, Bru JP, Therby A, Bouhour D, Dénes E, Debard A, Chirouze C, Fèvre K, Dupon M, Aegerter P, Mulleman D (2015) Antibiotic treatment for 6 weeks versus 12 weeks in patients with pyogenic vertebral osteomyelitis: an open-label, non-inferiority, randomised, controlled trial. Lancet 385(9971):875–882. 10.1016/s0140-6736(14)61233-2 25468170 10.1016/S0140-6736(14)61233-2

[10] Senneville E, Melliez H, Beltrand E, Legout L, Valette M, Cazaubiel M, Cordonnier M, Caillaux M, Yazdanpanah Y, Mouton Y (2006) Culture of percutaneous bone biopsy specimens for diagnosis of diabetic foot osteomyelitis: concordance with ulcer swab cultures. Clin Infect Dis 42(1):57–62. 10.1086/498112 16323092 10.1086/498112

[11] Zhou AY, Muir L, Harris J, Herrick AL (2014) The impact of magnetic resonance imaging in early diagnosis of hand osteomyelitis in patients with systemic sclerosis. Clin Exp Rheumatol 32(6 Suppl 86):S–232 25372805

[12] Haque A, Wyman M, Dargan D, Hughes M, Musson R, Caddick J, Giblin V (2021) Hand Osteomyelitis in patients with secondary Raynaud Phenomenon. J Clin Rheumatol 27(8s):S342–s345. 10.1097/rhu.0000000000001621 33337814 10.1097/RHU.0000000000001621

[13] The University of Tokyo Hospital Scleroderma Center < https://dermatology.m.u-tokyo.ac.jp/top/about-dermatology/speciality/ssc/ [Accessed 14 July 2024]

[14] van den Hoogen F, Khanna D, Fransen J, Johnson SR, Baron M, Tyndall A, Matucci-Cerinic M, Naden RP, Medsger TA Jr., Carreira PE, Riemekasten G, Clements PJ, Denton CP, Distler O, Allanore Y, Furst DE, Gabrielli A, Mayes MD, van Laar JM, Seibold JR, Czirjak L, Steen VD, Inanc M, Kowal-Bielecka O, Müller-Ladner U, Valentini G, Veale DJ, Vonk MC, Walker UA, Chung L, Collier DH, Ellen Csuka M, Fessler BJ, Guiducci S, Herrick A, Hsu VM, Jimenez S, Kahaleh B, Merkel PA, Sierakowski S, Silver RM, Simms RW, Varga J, Pope JE (2013) 2013 classification criteria for systemic sclerosis: an American college of rheumatology/European league against rheumatism collaborative initiative. Ann Rheum Dis 72(11):1747–1755. 10.1136/annrheumdis-2013-204424 24092682 10.1136/annrheumdis-2013-204424

[15] Khanna D, Furst DE, Clements PJ, Allanore Y, Baron M, Czirjak L, Distler O, Foeldvari I, Kuwana M, Matucci-Cerinic M, Mayes M, Medsger T Jr., Merkel PA, Pope JE, Seibold JR, Steen V, Stevens W, Denton CP (2017) Standardization of the modified Rodnan skin score for use in clinical trials of systemic sclerosis. J Scleroderma Relat Disord 2(1):11–18. 10.5301/jsrd.5000231 28516167 10.5301/jsrd.5000231 PMC5431585

[16] Giuggioli D, Manfredi A, Colaci M, Lumetti F, Ferri C (2012) Scleroderma digital ulcers complicated by infection with fecal pathogens. Arthritis Care Res (Hoboken) 64(2):295–297. 10.1002/acr.20673 22012860 10.1002/acr.20673

[17] Veve MP, Mercuro NJ, Sangiovanni RJ, Santarossa M, Patel N (2022) Prevalence and predictors of Pseudomonas aeruginosa among hospitalized patients with Diabetic Foot infections. Open Forum Infect Dis 9(7):ofac297. 10.1093/ofid/ofac297 35873292 10.1093/ofid/ofac297 PMC9301575

[18] Tone A, Nguyen S, Devemy F, Topolinski H, Valette M, Cazaubiel M, Fayard A, Beltrand É, Lemaire C, Senneville É (2015) Six-week versus twelve-week antibiotic therapy for nonsurgically treated diabetic foot osteomyelitis: a multicenter open-label controlled randomized study. Diabetes Care 38(2):302–307. 10.2337/dc14-1514 25414157 10.2337/dc14-1514

[19] Iranparvar M, Arzanlou M, Afrouzeh E (2019) Comparison of the efficacy of six-week versus twelve-week antibiotic therapy for the treatment of nonsurgical diabetic foot osteomyelitis. Int Med 1(5). 10.5455/im.53372

[20] Cortes-Penfield NW, Armstrong DG, Brennan MB, Fayfman M, Ryder JH, Tan TW, Schechter MC (2023) Evaluation and management of diabetes-related foot infections. Clin Infect Dis 77(3):e1–e13. 10.1093/cid/ciad255 37306693 10.1093/cid/ciad255 PMC10425200

[21] Williams AA, Carl HM, Lifchez SD (2018) The Scleroderma Hand: manifestations of Disease and Approach to Management. J Hand Surg Am 43(6):550–557. 10.1016/j.jhsa.2018.03.021 29691079 10.1016/j.jhsa.2018.03.021

[22] Pope JE, Denton CP, Johnson SR, Fernandez-Codina A, Hudson M, Nevskaya T (2023) State-of-the-art evidence in the treatment of systemic sclerosis. Nat Rev Rheumatol 19(4):212–226. 10.1038/s41584-023-00909-5 36849541 10.1038/s41584-023-00909-5 PMC9970138

[23] Ingegnoli F, Ughi N, Mihai C (2018) Update on the epidemiology, risk factors, and disease outcomes of systemic sclerosis. Best Pract Res Clin Rheumatol 32(2):223–240. 10.1016/j.berh.2018.08.005 30527428 10.1016/j.berh.2018.08.005

[24] Matucci-Cerinic M, Denton CP, Furst DE, Mayes MD, Hsu VM, Carpentier P, Wigley FM, Black CM, Fessler BJ, Merkel PA, Pope JE, Sweiss NJ, Doyle MK, Hellmich B, Medsger TA Jr., Morganti A, Kramer F, Korn JH, Seibold JR (2011) Bosentan treatment of digital ulcers related to systemic sclerosis: results from the RAPIDS-2 randomised, double-blind, placebo-controlled trial. Ann Rheum Dis 70(1):32–38. 10.1136/ard.2010.130658 20805294 10.1136/ard.2010.130658 PMC3002766

