# Supplementary material for: Characteristics and management of systemic sclerosis-related osteomyelitis: a retrospective cohort study
Source: Rheumatol Int. 2025 Mar 12;45(4):73. doi: 10.1007/s00296-025-05815-5 (PMC11903529; doi:10.1007/s00296-025-05815-5)
Supplement: Supplementary file 2 — Supplementary Material 2 [file 296_2025_5815_MOESM2_ESM.pdf]

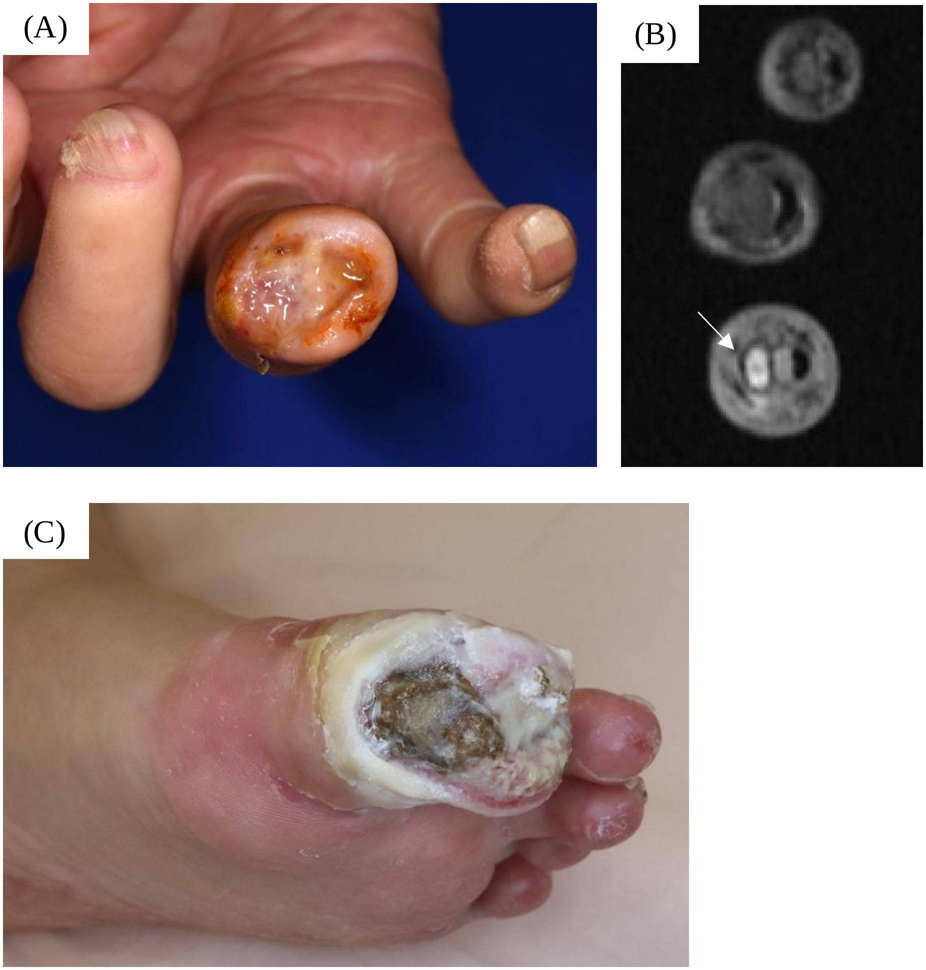


**Online Resource 2. Representing clinical manifestations and magnetic resonance imaging findings of systemic sclerosis-related osteomyelitis.**

A representative skin ulcer accompanied by osteomyelitis in the 4th finger (A) which showed high signal intensity in the middle phalanx on the fat-suppressed T2 sequence of magnetic resonance imaging (B, white arrow). A representative skin ulcer accompanied by osteomyelitis in the toe (C).
