# Supplementary material for: Characteristics and management of systemic sclerosis-related osteomyelitis: a retrospective cohort study
Source: Rheumatol Int. 2025 Mar 12;45(4):73. doi: 10.1007/s00296-025-05815-5 (PMC11903529; doi:10.1007/s00296-025-05815-5)
Supplement: Supplementary file 3 — Supplementary Material 3 [file 296_2025_5815_MOESM3_ESM.pdf]

Online Resource 3. The reliability between local clinical findings and serum C-reactive protein levels over the course in patients with initially elevated serum C-reactive protein levels (n = 23)

**Variables**	**Elevated CRP level**	**Normal CRP level**	**Kappa statistic**
**Local inflammation**			
3 months after the initiation of treatment (n = 18)[a]			0.31
Resolved	7	7	
Not resolved	4	0	
6 months after the initiation of treatment (n = 17)[b]			0.07
Resolved	10	6	
Not resolved	1	0	
12 months after the initiation of treatment (n = 12)[c]			0
Resolved	8	4	
Not resolved	0	0	
**Wound closure**			
3 months after the initiation of treatment (n = 18)[a]			0.06
Achieved	1	1	
Not achieved	10	6	
6 months after the initiation of treatment (n = 17)[b]			0.06
Achieved	3	2	
Not achieved	8	4	
12 months after the initiation of treatment (n = 12)[c]			0
Achieved	4	2	
Not achieved	4	2	

Note:

Abbreviations: CRP, C-reactive protein

[a]Data pertinent to 18 patients were available at 3 months post-treatment initiation, after excluding three who died and two who were transferred to other hospitals.

[b]Data pertinent to 17 patients were available at 6 months post-treatment initiation, after

excluding three who died and three who were transferred to other hospitals.

[c]Data pertinent to 12 patients were available at 12 months post-treatment initiation, after excluding four who died and seven who were transferred to other hospitals.
