# Supplementary material for: Characteristics and management of systemic sclerosis-related osteomyelitis: a retrospective cohort study
Source: Rheumatol Int. 2025 Mar 12;45(4):73. doi: 10.1007/s00296-025-05815-5 (PMC11903529; doi:10.1007/s00296-025-05815-5)
Supplement: Supplementary file 4 — Supplementary Material 4 [file 296_2025_5815_MOESM4_ESM.pdf]

Supplementary Table 1. The autoantibody profiles of study patients with positive systemic sclerosis-related autoantibodies

**Autoantibodies**	**Overall cohort (*n* = 2,126)**	**Patients without osteomyelitis (*n* = 2,079)**	**Patients with osteomyelitis (*n* = 47)**	***P* value**
Anti-centromere antibody	1051 (49.4)	1,026 (49.4)	25 (53.2)	0.60
Anti-U1RNP antibody	715 (33.6)	701 (33.7)	14 (29.8)	0.57
Anti-topoisomerase I antibody	428 (20.1)	411 (19.8)	17 (36.2)	< 0.01
Anti-RNA polymerase III antibody	66 (3.1)	65 (3.1)	1 (2.1)	1.00

Note:

Data are presented as a number (%) unless otherwise specified.

Abbreviations: U1RNP, U1-ribonucleoprotein
